# Increased organic fertilizer application and reduced chemical fertilizer application affect the soil properties and bacterial communities of grape rhizosphere soil

**DOI:** 10.1038/s41598-020-66648-9

**Published:** 2020-06-12

**Authors:** Linnan Wu, Yu Jiang, Fengyun Zhao, Xiufeng He, Huaifeng Liu, Kun Yu

**Affiliations:** 1 Department of Horticulture, Agriculture College, Shihezi University, Shihezi, 832003 P.R. China; 2 Key Laboratory of Special Fruit and vegetable Cultivation Physiology and Germplasm Resources Utilization of Xinjiang Production and Construction Crops, Shihezi University, Shihezi, 832003 P.R. China

**Keywords:** Plant development, Bacteria, Microbial communities

## Abstract

Increasing organic fertilizer application can improve the sustainability of soil productivity, but the effects of increased organic fertilizer application with reduced chemical fertilizer application over different time periods on chemical properties and bacterial community of grape rhizosphere soil in an arid region are not clear. In this study, three years of fixed-point field tests were used to compare the effects of various fertilization treatments on the soil properties and bacterial community in the grape rhizosphere. The results showed that (1) T1 and T2 significantly increased SOM, AN, AP and AK contents in grape rhizosphere soil. TN, TP and TK contents in grape leaves of T2 were the highest of those in five fertilization treatments. (2) The abundances of *Proteobacteria* and *Bacteroidetes* phyla and especially of *Arthrobacter*, *Pseudomonas*, *Nitrosopira* and *Bacillus* genera were higher in T2 than in the other samples. (3) SOM, AP and AN contents in soil were the main factors affecting soil bacterial community and mineral element contents in grape leaves and roots according to an RDA analysis. In summary, the application of organic fertilizer with reduced chemical fertilizer for two years had the greatest impact on the soil properties and bacterial community of the grape rhizosphere soil.

## Introduction

Soil microbes play an important role in maintaining soil productivity through biochemical processes such as litter decomposition and nutrient recycling^1^. The soil microbial community is an important factor influencing plant health because plant disease resistance mainly relies on the rhizosphere microbial community^2^. Microbes are regarded as early warning indicators of soil quality because of their rapid response and sensitivity to environmental changes^3^. Soil properties are greatly influenced by agronomic practices such as tillage, irrigation, and fertilization^4^. Fertilization is widely used worldwide to improve soil fertility. Excessive fertilizer application aggravates the decline of soil organic matter and fertility and accelerates soil acidification^5^. Soil properties affect the community structure of soil microbes^6^. Fertilization influences soil microbial diversity through direct effects on the soil nutrient content^7^. Soil microbial communities are sensitive to fertilization and their responses to manure and/or mineral fertilizers in soils have been well studied over the past several years^8,9^. The long-term application of nitrogen fertilizer or nitrogen fertilizer in combination with other mineral fertilizers affects the nitrogen cycle and related bacterial populations^10^. Repeated overuse of chemical fertilizer can have a negative effect on soil quality and soil microbial community structure. The long-term application of chemical fertilizers can significantly reduce soil pH which is closely associated with decreased bacterial diversity and significant changes in bacterial community composition, livestock manures can prevent soil acidification and its effects on soil bacteria^11^. Geisseler and Scow found that with fertilizer application, the soil microbial biomass increased by 15.1% compared with that under non-fertilized conditions, but fertilizer application tended to decrease the soil microbial biomass at pH <5 and promote soil microbial biomass accumulation at high pH levels^12^. Organic fertilizers have a greater impact on the microbial community structure than chemical fertilizers^13^. Xiong *et al*. found that the application of organic fertilizers reduced the occurrence of soil diseases and remodelled the structure and function of the soil bacterial community^14^. Organic fertilizers, such as animal manure, plant residues and composted organic matter, can change the soil bacterial community structure and activity and affect the abundance of the N-cycling-related microbiome^15,16^. Specific bacteria in the plant rhizosphere benefit plant growth by using available N, P and K^3^. Ammonia oxidation is the primary step in the oxidation of ammonia to nitrate via nitrite, and it is therefore central to the global nitrogen cycle^17^. Mineral fertilizers and organic manure affect the community structure of ammonia-oxidizing bacteria (AOB) in soils^18^. All known terrestrial AOB belong to a monophyletic group within the β-subclass of *Proteobacteria*, and the currently accepted classification recognizes only two genera within this group, *Nitrosospira* and *Nitrosomonas*^19^. *Nitrosospira* in soil can improve fertilizer N use efficiency in plant^18^. Some *Arthrobacter* strains are able to promote plant growth^20^ and restrain plant pathogenic bacteria and fungi^21^. *Pseudomonas* and *Bacillus* genera are the dominant phosphate-solubilizing bacteria (PSB) in agricultural soils^22^. PSB can transform insoluble phosphorus, which is difficult for plants to absorb and utilize, into available forms and improve the soil phosphorus utilization efficiency.

Xinjiang has the largest area of grape cultivation in China, at 149,000 hm^2^, which accounts for 18.4% of the total area of grape cultivation in China^23^. In recent years, due to the large-scale application of drip irrigation and chemical fertilizers, secondary salinization, decreased soil fertility and environmental pollution in the grape soil have become increasingly prominent^24^. Previous studies have shown that a combination of organic fertilizer and chemical fertilizer could improve fruit quality, change the soil properties, and improve the fruit rhizosphere environment^25^. However, there are few reports on the persistent effects of organic and chemical fertilizers on soil properties or on rhizosphere microbial communities in arid regions. For these reasons, this study used ‘Summer Black’ grapes as research material, through three years of fixed-point field tests to compare the effects of various fertilization treatments (no fertilizer, a typical chemical fertilizer, increased organic fertilizer and reduced chemical fertilizer for one year, two years and three years) on the soil properties and the grape rhizosphere bacterial community structure. Our aim was to provide a theoretical basis for the scientific fertilization of grapes in the field.

## Results

### Effects of different fertilizer treatments on grape rhizosphere soil propertie

Table 1 shows that the SOM, AN, AP and AK contents in the rhizosphere soil were the highest in T2 and the lowest in CK of the five different fertilizer treatments on day 15 after anthesis. The SOM and AK contents in the soils treated with increased organic fertilizer and reduced chemical fertilizer (T1, T2, and T3) were higher than those of CK and T0. The content of AN in the rhizosphere soil of T0 was significantly lower than that of T2 (*P* < 0.05); however, there was no significant difference in AN content between T3 and T0 (*P* > 0.05). The AP content in the rhizosphere soil of T0 was significantly lower than that of T2 and T3 by 5.86% and 2.79%, respectively (*P* < 0.05), but the difference in AP content between T0 and T1 was not significant (*P* > 0.05). The pH in the rhizosphere soils treated with organic fertilizer and chemical fertilizer (T1, T2, and T3) were significantly lower than that in CK and T0 (*P* < 0.05). The soil conductivity in T2 and T3 were significantly lower than that in CK and T0 (*P* < 0.05), and soil conductivity in T2 decreased by 31.10% and 24.24% compared with CK and T0, respectively. There was no significant difference in soil conductivity between T1 and T0 (*P* > 0.05) (Table 1). The SOM, AN, AP and AK contents in the rhizosphere soil were the highest in M-T1 and the lowest in M-CK of the five different fertilizer treatments on day 75 after anthesis. The SOM, AN and AP contents in M-T1 soil were 8.40%, 5.67% and 5.82% higher than those in M-T2 soil, respectively. The soil pH in M-T2 decreased by 1.73% and 1.48% compared with that in M-CK and M-T0. Soil pH in M-T1 was lower than that in M-CK and M-T0 (*P* > 0.05). The soil conductivity in M-T2 was the lowest, followed by that in M-T3, and the soil conductivity in M-T3 was 36.77% and 18.22% lower than that in M-CK and M-T0 (*P* < 0.05) (Table 1).

**Table 1:** Effects of different fertilizer treatments on the properties of grape rhizosphere soil on days 15 and 75 after anthesis. Values are means ± standard errors (n = 3).

Number of days after anthesis (d)	Treatment	SOM (g/kg)	AN (mg/kg)	AP (mg/kg)	AK (mg/kg)	pH	Conductivity (us/cm)
	CK	31.58 ± 1.42d	48.64 ± 1.08d	34.36 ± 0.82d	148.19 ± 0.89d	8.13 ± 0.03a	0.254 ± 0.009a
	T0	44.20 ± 1.14c	64.74 ± 1.27b	78.47 ± 1.46c	163.58 ± 1.55c	8.15 ± 0.10a	0.231 ± 0.017b
15 d	T1	53.07 ± 1.00b	59.39 ± 0.80c	78.75 ± 1.11bc	170.46 ± 1.24c	8.07 ± 0.02b	0.203 ± 0.026bc
	T2	56.95 ± 1.03a	69.26 ± 1.10a	83.36 ± 1.12a	218.18 ± 1.63a	8.09 ± 0.01b	0.175 ± 0.008c
	T3	53.28 ± 0.70b	64.61 ± 1.00b	80.72 ± 1.24b	210.25 ± 1.44b	8.09 ± 0.01b	0.191 ± 0.008c
	M-CK	35.25 ± 1.34d	41.89 ± 1.32d	41.17 ± 1.17d	136.89 ± 1.53d	8.10 ± 0.02a	0.291 ± 0.029a
	M-T0	51.08 ± 1.03c	61.94 ± 1.88c	68.37 ± 0.92c	262.86 ± 2.32b	8.09 ± 0.03a	0.225 ± 0.006b
75 d	M-T1	70.85 ± 1.64a	70.52 ± 0.96a	82.36 ± 1.45a	282.14 ± 1.60a	8.04 ± 0.01ab	0.202 ± 0.007c
	M-T2	65.36 ± 0.75b	66.73 ± 1.40b	77.83 ± 0.93b	267.83 ± 1.34b	7.97 ± 0.04b	0.171 ± 0.004c
	M-T3	63.14 ± 1.22b	64.06 ± 0.92c	68.12 ± 1.45c	251.15 ± 1.60c	8.02 ± 0.07b	0.184 ± 0.009c

Different letters indicate significant differences (*P* < 0.05) between two treatments. SOM: soil organic matter; AN: alkali-hydrolysed nitrogen; AP: available phosphorus; AK: available potassium.

### Effects of different fertilizer treatments on the total N, total P and total K of grape roots and leaves

On day 15 after anthesis, the TN, TP and TK contents of grape roots were the highest of the five different fertilizer treatments in T2 and the lowest in CK (Table 2). The content of TN in the grape roots of T2 decreased by 73.70% and 10.50% compared with that in CK and T0, respectively. The content of TP in the grape roots of T2 was significantly higher than that of CK and T0 (*P* < 0.05), and the TP content of T0 was higher than that of T1 and T3 (*P* > 0.05). The TK content in the grape roots of T2 increased by 145.83% and 37.21% compared with that of CK and T0, and the TK content of T0 was significantly lower than that of T1 and T3 (*P* < 0.05). The contents of TN, TP and TK in grape leaves were the highest of five treatments in T2 and the lowest in CK. The contents of TN, TP and TK in grape leaves of T2 were significantly higher than those of CK and T0 (*P* < 0.05). The contents of TN and TP in the grape leaves of T0 were significantly higher than those of T1 and T3 (*P* < 0.05). The TK content in the grape leaves of T0 was significantly higher than that of T1 and T3 (*P* < 0.05).

**Table 2:** Effects of different fertilizer treatments on the total N, total P and total K contents of grape roots and leaves on days 15 and 75 after anthesis. Values are means ± standard errors (n = 3).

Number of days after anthesis (d)	Treatment	Roots			Leaves		
		TN	TP	TK	TN	TP	TK
	CK	4.9 ± 0.31e	0.10 ± 0.08d	0.24 ± 0.03d	8.38 ± 0.17e	0.25 ± 0.04d	1.24 ± 0.02d
	T0	7.70 ± 0.42b	0.36 ± 0.13b	0.43 ± 0.03c	10.73 ± 0.54b	0.44 ± 0.05ab	1.58 ± 0.02ab
15 d	T1	6.25 ± 0.22d	0.29 ± 0.05c	0.50 ± 0.03b	9.02 ± 0.10d	0.37 ± 0.02c	1.42 ± 0.04c
	T2	8.51 ± 0.26a	0.42 ± 0.12a	0.59 ± 0.03a	11.77 ± 0.45a	0.48 ± 0.02a	1.62 ± 0.08a
	T3	6.99 ± 0.22c	0.31 ± 0.02bc	0.46 ± 0.04bc	9.75 ± 0.27c	0.41 ± 0.03c	1.52 ± 0.06b
	M-CK	4.53 ± 0.28c	0.10 ± 0.23d	0.21 ± 0.02c	7.08 ± 0.20c	0.47 ± 0.04d	1.33 ± 0.09c
	M-T0	5.25 ± 0.33b	0.40 ± 0.12c	0.64 ± 0.04b	8.86 ± 0.72b	0.63 ± 0.03c	1.56 ± 0.04b
75 d	M-T1	6.84 ± 0.12a	0.48 ± 0.04b	0.71 ± 0.04a	9.38 ± 0.42b	0.69 ± 0.03bc	1.65 ± 0.02ab
	M-T2	6.77 ± 0.08a	0.56 ± 0.03a	0.76 ± 0.05a	10.36 ± 0.33a	0.75 ± 0.03a	1.72 ± 0.03a
	M-T3	5.50 ± 0.39b	0.42 ± 0.02c	0.75 ± 0.05a	9.32 ± 0.31b	0.74 ± 0.04ab	1.57 ± 0.04b

Different letters indicate significant differences (*P* < 0.05) between two treatments. TN: total nitrogen; TP: total phosphorus; TK: total potassium.

On day 75 after anthesis, the TN, TP and TK contents in the grape roots of M-CK were the lowest in all treatments, whereas the TN content in grape roots treated with increased organic fertilizer and reduced chemical fertilizer (M-T1, M-T2, and M-T3) were higher than that of M-T0 (*P* > 0.05), and the difference between M-T0 and M-T3 was not significant (*P* > 0.05). The TP content in the grape roots of M-T2 was significantly higher than that of M-CK and M-T0 by 460.00% and 16.00%, respectively. The TP content of M-T1 was significantly higher than that of M-T0 (*P* < 0.05), but there was no significant difference in TP content between M-T3 and M-T0 (*P* > 0.05). The TK contents in grape roots treated with increased organic fertilizer and reduced chemical fertilizer (M-T1, M-T2, and M-T3) were significantly higher than those of M-CK and M-T0 (*P* < 0.05), and there was no significant difference in TK content among M-T1, M-T2 and M-T3. The contents of TN, TP and TK in grape leaves were the highest of five different fertilizer treatments in M-T2 and the lowest in M-CK. The TN content in the grape leaves of M-T2 was significantly higher than those of M-CK and M-T0 (*P* < 0.05). The TP contents in grape leaves of M-T2 and M-T3 were significantly higher than those of M-CK and M-T0 (*P* < 0.05), and the difference in leaves TP content between M-T1 and M-T0 was not significant (*P* > 0.05). The TK content in the grape leaves of M-T2 was significantly higher than those of M-CK and M-T0 by 29.32% and 10.53%, respectively (*P* < 0.05) (Table 2).

### Effects of different fertilizer treatments on bacterial richness and diversity in grape rhizosphere soil

**Figure 1:**
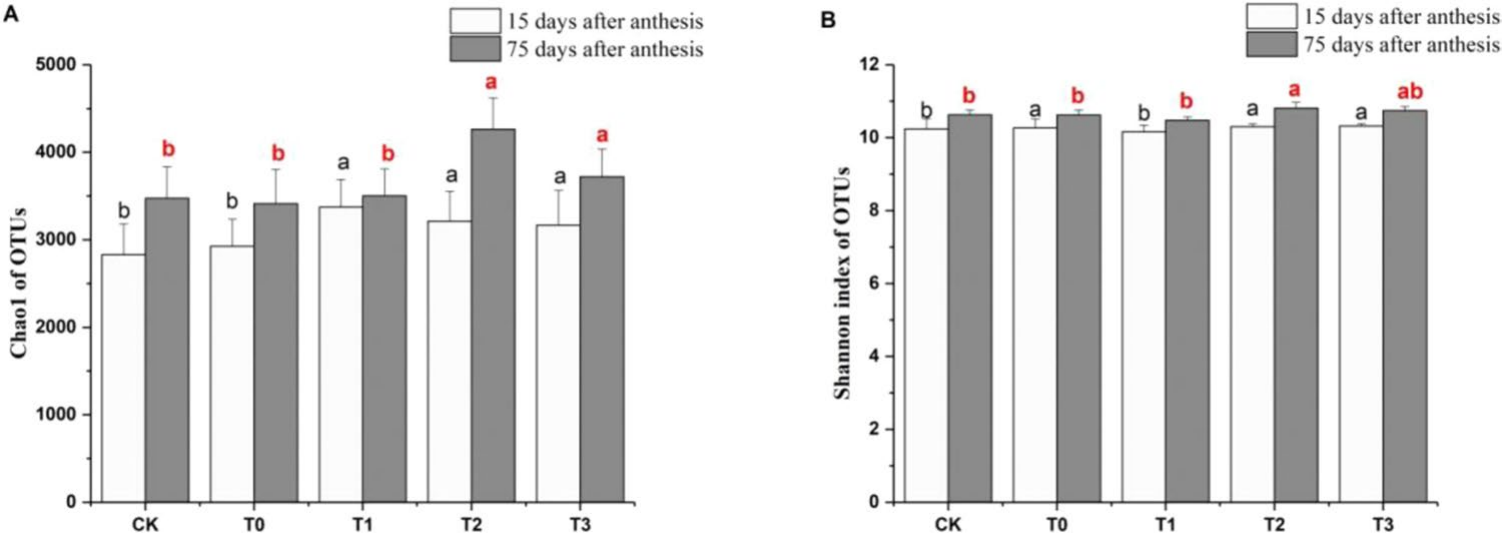
Effects of different fertilizer treatments on the Chao1 and Shannon indexes of bacteria in grape rhizosphere soil on days 15 and 75 after anthesis.

On day 15 after anthesis, the soil bacterial richness (estimated by Chao1) of the increased organic fertilizer and reduced chemical fertilizer treatments (T1, T2, and T3) were significantly greater than that of CK and T0 (*P* < 0.05), but there was no significant difference in bacterial richness among T1, T2 and T3 (*P* > 0.05). The Chao1 of T1 was higher than that of CK and T0 by 19.26% and 15.27%, respectively. On day 75 after anthesis, the Chao1 of M-T2 was significantly (22.67% and 24.90%) higher than that of M-CK and M-T0 (*P* < 0.05) (Fig. 1A). The soil bacterial diversity (estimated by the Shannon index) of T2 and T3 were significantly higher than that of CK, by 0.59% and 0.78%, respectively. The Shannon index of T0 was significantly higher than that of T1. The Shannon index of M-T2 was significantly higher than that of M-CK and M-T0, by 1.69% and 1.78%, respectively (*P* < 0.05) (Fig. 1B).

### Effects of different fertilizer treatments on bacterial communities at the phylum level in grape rhizosphere soil

**Figure 2:**
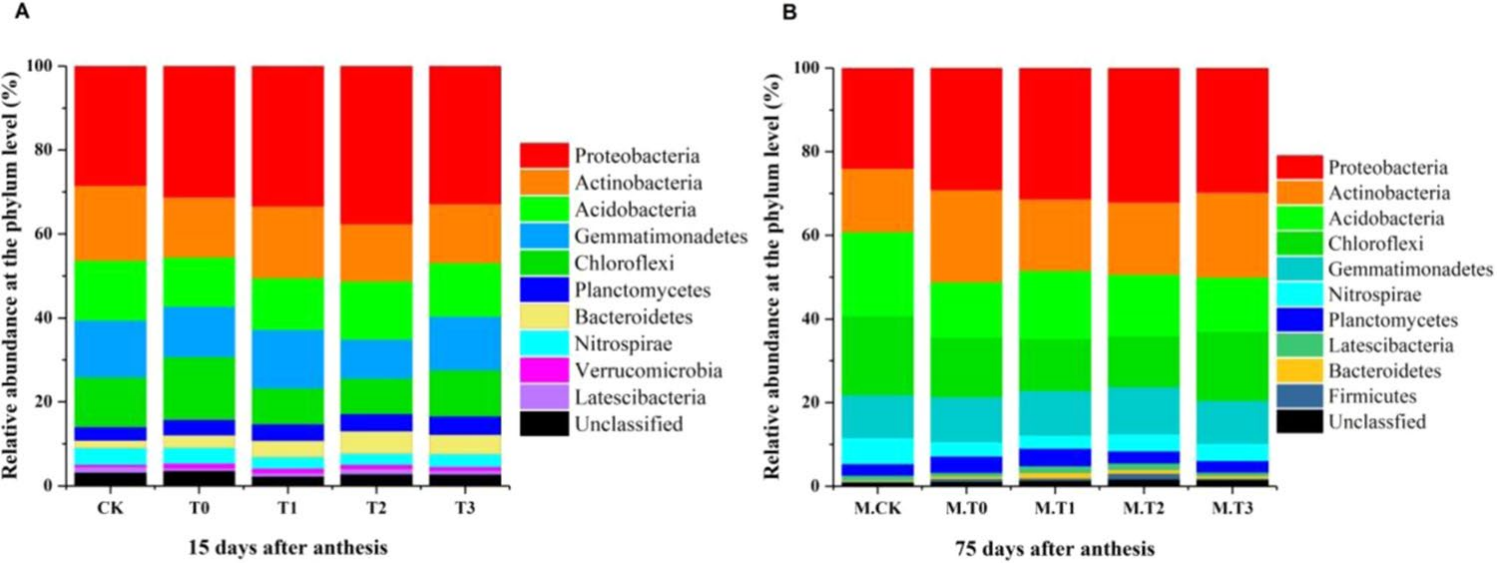
Effects of different fertilizer treatments on bacterial communities at the phylum level on days 15 and 75 after anthesis. (Phyla with relative abundances of less than 1% of the total composition in the libraries were considered unclassified).

Phyla with an average abundance greater than 1% in at least one soil sample were defined as dominant and are assessed in the following analysis. The soil samples had similar bacterial compositions, but some variations were observed in the distribution of each phylum. *Proteobacteria*, *Actinobacteria*, *Acidobacteria*, *Gemmatimonadetes* and *Chloroflexi* were the dominant phyla, demonstrating >10% relative abundance in at least one rhizosphere soil sample. Another five predominant phyla were *Planctomycetes*, *Bacteroidetes*, *Nitrospirae*, *Verrucomicrobia* and *Latescibacteria* on days 15 and 75 after anthesis (Fig. 2). The relative abundance of *Chloroflexi* in T0 was significantly lower than that of CK on days 15 and 75 after anthesis (*P* < 0.05), and the relative abundance of *Chloroflexi* in soils treated with increased organic fertilizer and reduced chemical fertilizer (T1, T2, and T3) was significantly lower than that in CK and T0 (*P* < 0.05) (Table 3). On day 15 after anthesis, the relative abundances of *Gemmatimonadetes* and *Bacteroidetes* were the highest in T2 and the lowest in CK in the five different fertilizer treatments. The relative abundances of *Gemmatimonadetes* in T2 and T3 were significantly higher than that in CK by 51.11% and 6.83%, respectively. The relative abundance of *Bacteroidetes* in T2 was significantly higher than that in CK, and the relative abundance of *Bacteroidetes* in T0 was higher than that in T1 and lower than that in T3 (*P* < 0.05).

**Table 3:** Effects of different fertilizer treatments on bacterial communities at the phylum level in grape rhizosphere soil. Values are means ± standard errors (n = 3). (Only bacterial phyla with significant differences in five treatments are shown).

Number of days after anthesis (d)		CK	T0	T1	T2	T3
15 d	Gemmatimonadetes	9.20 ± 3.21b	12.73 ± 1.37ab	12.00 ± 0.61ab	13.90 ± 2.48a	13.60 ± 1.25a
	Chloroflexi	14.80 ± 3.40a	11.70 ± 0.96a	8.57 ± 0.75b	8.60 ± 1.35b	10.93 ± 0.45b
	Bacteroidetes	1.87 ± 1.10b	3.97 ± 1.25ab	3.03 ± 0.45ab	5.33 ± 3.23a	4.50 ± 1.11ab
75 d		M-CK	M-T0	M-T1	M-T2	M-T3
	Proteobacteria	23.93 ± 1.53b	29.67 ± 3.26ab	29.10 ± 1.01ab	32.00 ± 2.65a	31.27 ± 5.61a
	Actinobacteria	22.07 ± 1.78a	17.33 ± 3.12ab	17.13 ± 3.47ab	15.27 ± 2.70b	20.33 ± 2.39ab
	Chloroflexi	18.97 ± 1.53a	16.53 ± 1.25a	14.23 ± 1.46b	12.26 ± 1.50b	12.50 ± 3.64b

Different letters indicate significant differences (*P* < 0.05) between twotreatments.

On day 75 after anthesis, the relative abundance of *Proteobacteria* was highest in M-T2 and lowest in M-CK of the five different fertilizer treatments. The relative abundance of *Actinobacteria* was the lowest in M-T2 and the highest in M-CK of the five different fertilizer treatments. The relative abundance of *Proteobacteria* in M-T2 and M-T3 were significantly higher than that in M-CK (*P* < 0.05), and the relative abundance of *Proteobacteria* in M-T0 was lower than that in M-T3 (*P* > 0.05). The relative abundance of *Actinobacteria* in M-T2 was significantly lower than that in M-CK by 30.81% (*P* < 0.05) (Table 3).

### Effects of different fertilizer treatments on N and P metabolism-related bacteria in grape rhizosphere soil at the genus level

On day 15 after anthesis, the relative abundance of *Nitrospira* in T2 was the highest of the five different fertilizer treatments and that in CK was the lowest. The abundance of *Nitrospira* in T2 was significantly higher than that in CK and T0 by 91.80% and 112.65%, respectively (*P* < 0.05) (Table 4). The relative abundances of *Pseudomonas* and *Arthrobacter* in T2 were the highest of the five different fertilizer treatments and that in CK was the lowest. The relative abundances of *Pseudomonas* in T2 increased by 277.84% and 140.83% compared with that in CK and T0, respectively. The relative abundance of *Arthrobacter* in T2 and T3 was significantly higher than that in CK and T0 (*P* < 0.05). The relative abundance of *Bacillus* in T2 increased by 105.74% and 74.96% compared with that in CK and T0 (*P* < 0.05) (Table 4).

**Table 4:** Effects of different fertilizer treatments on bacteria related to the metabolism of N and P in the grape rhizosphere soil at the genus level. Values are means ± standard errors (n = 3).

Number of days after anthesis (d)	Treatment	Nitrospira	Pseudomonas	Arthrobacter	Bacillus
	CK	5.61 ± 2.51b	3.34 ± 1.65b	2.18 ± 1.34b	5.40 ± 2.91b
	T0	5.06 ± 1.97b	5.24 ± 3.53ab	4.42 ± 1.14b	6.35 ± 0.55b
15 d	T1	4.51 ± 1.79b	1.90 ± 0.83b	5.00 ± 3.80b	7.30 ± 1.98ab
	T2	10.76 ± 1.99a	12.62 ± 5.55a	11.19 ± 2.70a	11.11 ± 3.10a
	T3	7.38 ± 3.19ab	10.24 ± 5.9ab	8.92 ± 2.86a	3.17 ± 1.46b
	M-CK	6.06 ± 0.47b	5.77 ± 1.66ab	3.42 ± 1.68b	4.13 ± 1.28b
	M-T0	5.23 ± 0.55b	5.29 ± 1.73ab	6.46 ± 2.52ab	5.73 ± 2.35b
75 d	M-T1	7.32 ± 1.37a	7.21 ± 5.83ab	6.39 ± 1.39ab	6.87 ± 4.10ab
	M-T2	7.58 ± 3.56a	11.86 ± 6.27a	8.76 ± 1.02a	8.71 ± 2.62a
	M-T3	7.15 ± 1.35ab	3.21 ± 0.28b	8.30 ± 2.17a	7.75 ± 3.09ab

Different letters indicated significant differences (*P* < 0.05) between two treatments.

On day 75 after anthesis, the relative abundance of *Nitrospira* in M-T2 was the highest of the five different fertilizer treatments and that in M-CK was the lowest. The abundance of *Nitrospira* in M-T2 was significantly higher than that in M-CK and M-T0 by 25.08% and 44.93%, respectively (*P* < 0.05) (Table 4). The relative abundances of *Pseudomonas* and *Arthrobacter* in M-T2 were the highest of the five different fertilizer treatments, and those in M-CK were the lowest. The relative abundance of *Pseudomonas* in M-T2 increased by 105.05% and 124.20% compared with that in M-CK and M-T0 (*P* < 0.05). The relative abundances of *Arthrobacter* in M-T2 and M-T3 were significantly higher than that in M-CK (*P* < 0.05). The relative abundance of *Arthrobacter* in the M-T2 was significantly higher than that in M-CK and M-T0, by 110.90% and 52.10%, respectively (*P* < 0.05) (Table 4).

### Relationship between grape rhizosphere soil properties, the bacterial community in grape rhizosphere soil and mineral element contents of grape roots and leaves

**Figure 3:**
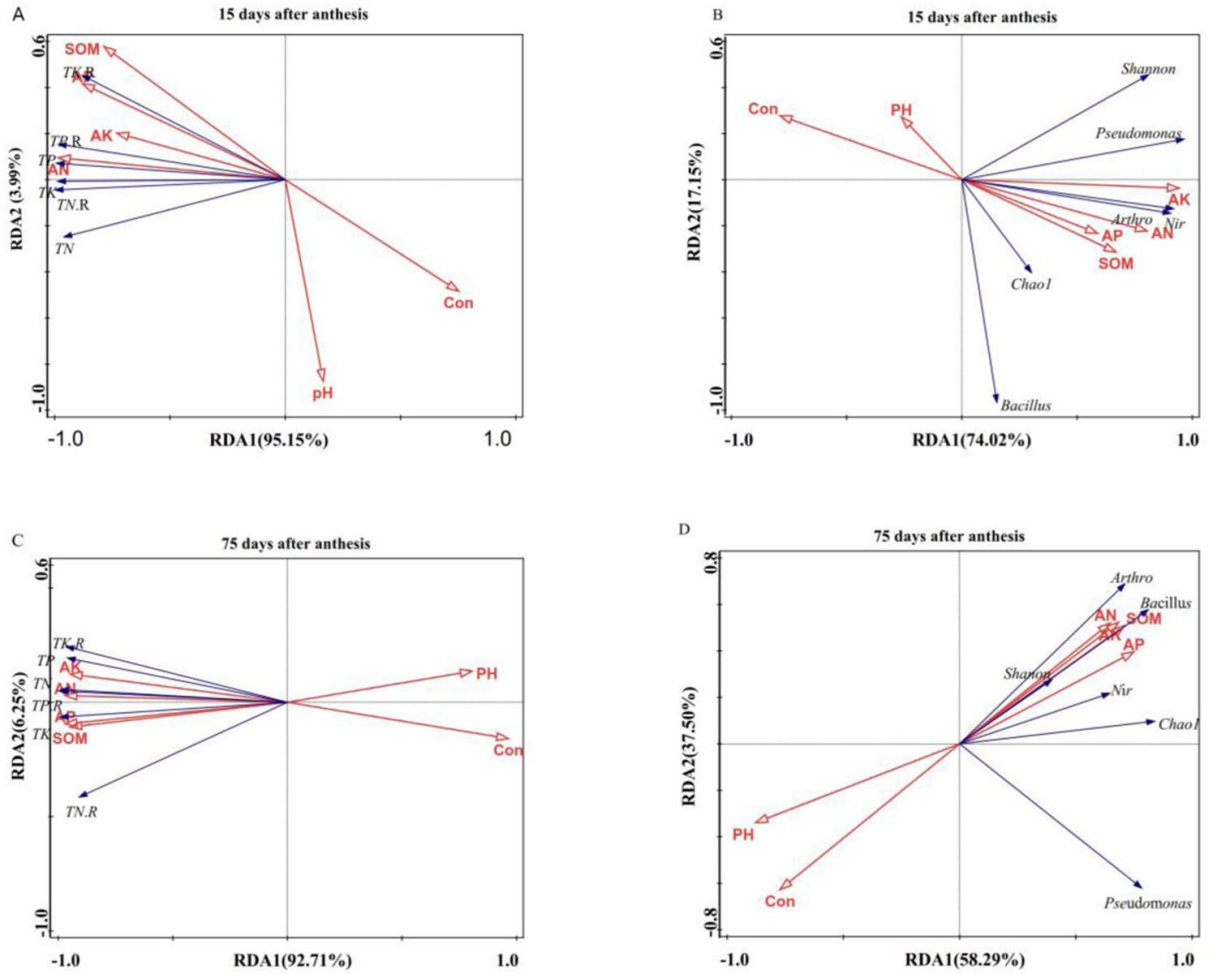
Redundancy analyses depicting the relationships among grape rhizosphere soil properties, the bacterial community in the grape rhizosphere soil and mineral element contents of grape roots and leaves. The relationships between the mineral element contents of grape roots and leaves and the grape rhizosphere soil properties (**A,C**). The relationships between the bacterial community in the grape rhizosphere soil and the grape rhizosphere soil properties (**B,D**). TN.R: total N in grape roots, TP.R: total P in grape roots, TK.R: total K in grape roots, TN: total N in grape leaves, Con: conductivity, Nir: *Nitrosopira*, Arthro: *Arthrobacter*.

The associations between grape rhizosphere soil properties and the mineral element contents of grape leaves and roots were analysed by RDA. pH and conductivity were negatively correlated with TN, TP, TK, TN.R, TP.R and TK.R on days 15 and 75 after anthesis. SOM, AN, AP and AK were positively correlated with TN, TP, TK, TN.R, TP.R and TK.R on days 15 and 75 after anthesis (Fig. 3A,C). On day 15 after anthesis, axis 1 and axis 2 explained 95.15% and 3.99% of the total variation, respectively (Fig. 3A). The mineral element contents of grape leaves and roots were significantly associated with SOM, AN and AP (*P* < 0.05). On day 75 after anthesis, axis 1 and axis 2 respectively explained 92.71% and 6.25% of the total variation (Fig. 3C). The mineral element contents of grape leaves and roots were significantly associated with SOM and AN (*P* < 0.05). Figure 3B,D show that the relationships between the grape rhizosphere soil properties and the microbial community composition in the grape rhizosphere soil and grape rhizosphere soil properties were considered environmental variables. The Chao1 and Shannon index values and the abundances of *Arthrobacter*, *Pseudomonas*, *Nitrosopira* and *Bacillus* were negatively correlated with pH and conductivity on days 15 and 75 after anthesis (Fig. 3B,D). The Chao1 and Shannon index values and the abundances of *Arthrobacter*, *Pseudomonas*, *Nitrosopira* and *Bacillus* were positively correlated with SOM, AN, AP and AK. The microbial community composition was significantly correlated with SOM and AP (*P* < 0.05).

## Discussion

In this study, the soil pH was significantly decreased by the increased organic fertilizer and reduced chemical fertilizer treatments compared to that in the typical chemical fertilizer and unfertilized treatments (Table 1). Intriguingly, increasing the amount of organic fertilizer and reducing the amount of chemical fertilizer did not stabilize or increase the soil pH but significantly decreased the soil pH, which disagreed with previous findings^26,27^. This discrepancy might be attributed to differences in the soil type, as proposed by Wei *et al*.^13^, who observed that organic fertilizer decreased the soil pH in alkaline soils but increased the soil pH in acidic soils. Our fertilization treatments also resulted in distinct soil compositions. The five different fertilization treatments showed significant differences in SOM content (Table 1). The SOM content in T2 was the highest of the five fertilizer treatments on day 15 after anthesis and that in T1 was the highest of the five fertilizer treatments on day 75 after anthesis. This was in line with previous studies in which the application of organic fertilizer increased the soil organic carbon content^28^. Some reports have shown that organic matter treatments with high levels of C directly increased the soil organic C content^29^. The AN content in the T0, T1, T2 and T3 fertilization treatments was higher than that in CK; AP and AK showed the same trends. Another previous study showed that the long-term combined application of chemical fertilizer and organic fertilizer significantly increased the contents of TN, AN and AP in the soil^30^. Garcia-Orenes *et al*. found that the application of organic fertilizer increased the contents of SOM, AK and AP^31^. In this experiment, we found that on days 15 and 75 after anthesis, the increased organic fertilizer with reduced chemical fertilizer treatments for one and two years led to the contents of SOM, AK and AP being significantly higher than those of unfertilized and typical chemical fertilization.

Zhao’s research showed that the combination of chemical fertilizer and organic fertilizer significantly increased the contents of N, P and K in apple leaves^32^. Tao *et al*. found that the combination of chemical and organic fertilizers increased cotton plant N and P uptake and fertilizer utilization efficiency^33^. The contents of TN, TP and TK in the grape leaves and roots treated with chemical and organic fertilizers were significantly higher than those in unfertilized leaves and roots, and T2 increased those parameters the most significantly (Table 2). This indicated that applying organic fertilizer and reducing chemical fertilizer application could improve nutrient absorption in grapes, and the effect of applying organic fertilizer and reducing chemical fertilizer for two years was greatest. This may be due to the organic fertilizers improving soil aeration and water retention in the graperhizosphere, which promotes the absorption of N, K and P by the grape roots.

Increasing or decreasing soil nutrients will change the soil bacterial abundance and diversity^34^. Yuan *et al*. found that organic fertilizer application significantly increased soil bacterial diversity and viability^35^. Sun *et al*. also showed that bacterial abundance was increased by the application of chemical fertilizer alone and the combined application of chemical and organic fertilizers^34^. In this study, the bacterial diversity under increased organic fertilizer and reduced chemical fertilizer for two years was significantly higher than that in the unfertilized and typical chemical fertilizer treatments. Most of the dominant bacteria in the soil were basically the same, including approximately 10 bacterial phyla^36^. *Proteobacteria*, *Actinobacteria*, *Acidobacteria*, *Gemmatimonadetes* and *Chloroflexi* were the five most abundant bacterial phyla in the soil samples on days 15 and 75 after anthesis (Fig. 2). Nitrification is the driving force of soil nitrogen cycling^4^. Nitrification, which is the oxidation of ammonia (NH_3_) to nitrite (NO_2_^−^), and then to nitrate (NO_3_^−^), is a key step within the N cycle. In agricultural systems, nitrification is associated with potential N losses from soils, either through the loss of NO_3_^−^ by leaching and/or through denitrification^4^. *Nitrospira* is an ammonia-oxidizing bacteria that contributes to soil nitrification^37^. Phosphorus is an indispensable nutrient for plant growth^22^. Unavailable forms of phosphorus in soil account for 95% of the total soil phosphorus, and plants cannot absorb and utilize these forms directly^22^. Phosphorus-solubilizing bacteria can transform insoluble phosphorus, which is difficult for plants to absorb and utilize, into available forms and improve the soil phosphorus utilization efficiency. Several studies have demonstrated that members of the *Pseudomonas* and *Bacillus* genera were the dominant phosphate-solubilizing bacteria (PSB) in agricultural soils. Kumar and Singh reported that the inoculation of vermicompost with the phosphate solubilizing *Pseudomonas striata* significantly increased the available P^38^. Bacterial strains belonging to the genera *Bacillus* and *Pseudomonas* have been isolated from the rhizosphere of various plants and are reported to improve plant growth^39^. In our study, the relative abundance of *Nitrospira* in the increased organic fertilizer and reduced chemical fertilizer for two years treatment (T2) was significantly higher than that in the unfertilized or typical chemical fertilizer treatments (CK and T0) (Table 4), similar to *Bacillus* and *Pseudomonas*. This indicated that grape rhizosphere soil treated with increased organic fertilizer and reduced chemical fertilizer for two years (T2) contained more available N and available P than the rhizosphere soil of the other treatments, which is beneficial for plant growth.

In our study, the RDA analysis showed that SOM and AP were the main environmental factors affecting the soil bacterial community structure in grape rhizosphere soil (Fig. 3). The microbial community composition was significantly correlated with SOM and AP in Xu’s study^3^. Nie also found that the fungal community composition was significantly correlated with SOM^40^. Pan’s study found that the bacterial community structure was significantly related to soil properties, such as SOM and pH^41^. Gu *et al*. found that the available N content in soil was positively correlated with the total N content in *Baren Apricot* leaves^42^. We found that the mineral element contents of the grape leaves and roots were significantly positively correlated with SOM, AN and AP in soil (*P* < 0.05). In summary, fertilization changed the chemical properties of the grape rhizosphere soil, which in turn affected the soil bacterial community structure, and ultimately affected the mineral element contents of the grape leaves and roots.

## Conclusions

In conclusion, this study showed that the different fertilization treatments resulted in different soil properties, bacterial diversity conditions and TN, TP and TK contents in grape roots and leaves. The T2 treatment had the highest AN, AP and SOM contents of all the treatments on day 15 after anthesis. The contents of TN, TP and TK in grape leaves and roots treated with increased organic fertilizer and reduced chemical fertilizer for two years (T2) were the highest of the five fertilizer treatments on days 15 and 75 after anthesis. The fertilizer treatments changed the abundance and diversity of bacteria and the relative abundances of *Nitrospira, Pseudomonas*, *Arthrobacter* and *Bacillus* in the rhizosphere soil of ‘Summer Black’ grapes. The relative abundances of *Nitrospira, Pseudomonas*, *Arthrobacter* and *Bacillus* were most significantly increased by increased organic fertilizer and reduced chemical fertilizer for two years. The SOM, AP and AN contents in soil were the main factors affecting the soil bacterial community and the mineral element content in grape leaves and roots according to the RDA analysis.

## Materials and Methods

### Experimental site and soil description

The field plot experiment was conducted at the Shihezi Agricultural Science Research Institute of Xinjiang Uygur Autonomous Region for three consecutive years from 2016 to 2018 (45°19′N, 86°03′E). The average annual temperature was between 6.5 and 7.2 °C, the annual precipitation was 225 mm, and the annual evaporation was 1342 mm. The soil was a sandy loam soil. The basic chemical properties of the 0.20–0.40 m soil were as follows: pH, 8.02; soil organic matter (SOM), 31.23 g/kg; total nitrogen, 135.32 mg/kg; alkali-hydrolysed nitrogen (AN), 51.11 mg/kg; total phosphorus (TP), 89.93 mg/kg; available phosphorus (AP), 32.95 mg/kg; total potassium (TK), 369.56 mg/kg; available potassium (AK), 130.35 mg/kg; and conductivity, 0.221 us/cm.

### Experimental design

The experiment used 7-year-old ‘Summer Black’ table grape plants as the research material. The grapes are planted in a north-south orientation, with a line spacing of 3 m and a plant spacing of 0.7 m. The grape trellis is a V-shaped scaffold, placed between the vines. The study used a randomized block design with three replicates of five treatments. Fifteen plots were used, and there were 7 well-grown, healthy grapes plants in each plot. The five treatments were (1) no fertilization for three consecutive years (CK), (2) typical chemical fertilization for three consecutive years (T0), (3) increased organic fertilizer and reduced chemical fertilizer for one year (T1), (4) increased organic fertilizer and reduced chemical fertilizer for two years (T2) and (5) increased organic fertilizer and reduced chemical fertilizer for three years (T3). The nutrient content of the fermented and decomposed cow manure was 2.48% (total nitrogen), 1.79% (total phosphorus) and 2.50% (total potassium). Organic fertilizer (cow manure) was applied in spring at depths of 25 cm and 30 cm from the main trunk of the grape plant(unilateral fertilization). The chemical fertilizers (drip application) used were urea (N: 46%), monoammonium phosphate (N: 12%, P_2_O_5_: 60%), and potassium sulfate (K_2_O: 50%) applied during the growing season. The chemical fertilizer was applied with water-fertilizer integrated device with a flow rate of 2.6 L/h and a working pressure of 0.05–0.1 MPa. The drip irrigation belt was located at 30 cm to the north and south sides of the grapes, just above the area where the organic fertilizer was applied. During the growing season, the fertilizer application times and field management practices for the five treatments were the same as those for conventional grapes^43^. The specific fertilizer dosage and application times of each treatment are shown in Table 5.

**Table 5:** Specific fertilizer application amounts for different treatments and years.

Treatment	2016				2017				2018			
	N (kg/hm^2^)	P_2_O_5_ (kg/hm^2^)	K_2_O (kg/hm^2^)	Manure (kg/hm^2^)	N (kg/hm^2^)	P_2_O_5_ (kg/hm^2^)	K_2_O (kg/hm^2^)	Manure (kg/hm^2^)	N (kg/hm^2^)	P_2_O_5_ (kg/hm^2^)	K_2_O (kg/hm^2^)	Manure (kg/hm^2^)
CK	0	0	0	0	0	0	0	0	0	0	0	0
T0	286.5	225	337.5	0	286.5	225	337.5	0	286.5	225	337.5	0
T1	286.5	225	337.5	0	286.5	225	337.5	0	243.5	191.3	286.9	2921.4
T2	286.5	225	337.5	0	243.5	191.3	286.9	2921.4	243.5	191.3	286.9	0
T3	243.5	191.3	286.9	2921.4	243.5	191.3	286.9	0	243.5	191.3	286.9	0

### Soil and plant sampling

The rhizosphere soil samples (20–40 cm underground) were collected with an auger on days 15 and 75 after anthesis (pre-swelling and mature stage). Soil samples were collected using the five-point sampling method. Five soil cores were selected from each plot, and then a composite sample was formed by mixing the soil cores from each plot. The soil samples were stored in aseptic sealed bags, sent to the laboratory and passed through a 2 mm sieve. Each sample was divided into two parts. One was dried in a cool place for chemical analysis, and the other stored at −80 °C and used for the determination of soil microorganisms properties. The grape roots and leaves were washed and dried.

### Soil and plant physicochemical properties

The pH, conductivity, organic matter, total nitrogen, alkaline-hydrolysed nitrogen, available phosphorus, total phosphorus and total potassium contents were measured using modified methods described by Bao *et al*. with modifications^44^.

### Soil DNA extraction, PCR amplification, and Illumina sequencing

The soil samples were extracted using an E.Z.N.A.kit and a soil DNA kit. The V4 gene fragment was amplified using the primers 520 F (5′-AYTGGYDTAAAGNG-3′) and 806 R (5′-TACNVGGTATCTAATCC-3′). To ensure the efficiency and accuracy of the amplification, efficient, high-fidelity enzymes were used for PCR. The PCR system (30 µL) included 15 µL Phusion Master Mix, 3 µL primers, and 10 µL DNA template (5~10 ng). The reactions were incubated at 98°C for 1 min, followed by 30 cycles of (98 °C for 10 s, 50°C for 30 s and 72 °C for 30 s). After that, the PCR products were mixed and purified. After purification, the library was constructed and sequenced. To distinguish the soil samples taken at 15 d after anthesis from those taken 75 d after anthesis, the soil samples at 75 d after anthesis were labelled M-CK, M-T0, M-T1, M-T2 and M-T3.

### Sequence data analysis and statistics

Based on the barcodes and PCR-amplified sequences, the sample data were separated from the downloaded data. The barcode and primer sequences were intercepted and spliced with FLASH^45^ to obtain high quality original data. The sequence data were processed via QIIME for quality control^46^, and chimeric sequences were further removed to yield the final dataset. The final valid sequences were clustered using UPARSE software. By default, 97% sequence similarity was used to cluster the sequences to obtain OTUs. The Greengenes database was used as a reference for OTU clustering and material classification analysis. A Venn diagram was used to compare the OTUs in the soil, a heat map was used to visualize the double cluster analysis of the samples, and the abundance and diversity of soil rhizosphere bacteria were estimated based on the Chao1 and Shannon indexes.

### Statistical analysis

Excel 2010 and SPSS 19.0 were used for statistical analysis. The significance level was set to *P* = 0.05. Redundancy analysis (RDA) was calculated by CANOCO version 5.0 toelucidate the relationships between the basic soil chemical properties and the soil bacterial parameters.
